# miRNAs regulate stem cell self-renewal and differentiation

**DOI:** 10.3389/fgene.2012.00191

**Published:** 2012-09-25

**Authors:** Zuoren Yu, Yuan Li, Huimin Fan, Zhongmin Liu, Richard G. Pestell

**Affiliations:** ^1^ Research Center for Translational Medicine, Key Laboratory of Arrhythmia, East Hospital, Tongji University School of MedicineShanghai, China; ^2^ Department of Cancer Biology, Kimmel Cancer Center, Thomas Jefferson UniversityPhiladelphia, PA, USA

**Keywords:** stem cell, microRNA, epigenetic modification, self-renewal

## Abstract

Stem cells undergo symmetric and asymmetric divisions to generate differentiated cells and more stem cells. The balance between self-renewal and differentiation of stem cells is controlled by transcription factors, epigenetic regulatory networks, and microRNAs (miRNAs). Herein the miRNA involvement in the regulation of stem cell self-renewal and differentiation is summarized. miRNA contribution to malignancy through regulating cancer stem cells is described. In addition, the reciprocal associations between miRNAs and epigenetic modifications in control of stem cell fate are discussed.

## INTRODUCTION

Stem cells are a small population of cells with the dual capacities for both self-renewal thereby producing more stem cells and differentiation thereby generating specialized cell types (Draper et al., 2004; Velasco-Velazquez et al., 2012). Through symmetric division, one stem cell divides into two new stem cells. Through asymmetric division, one stem cell divides into another stem cell and one differentiating cell. Stem cells have been identified from embryos and various adult tissues, as well as bone marrow and blood. Embryonic stem (ES) cells are pluripotent and give rise to diverse cell types and tissues. Autologous adult stem cell transplantation is considered as one of the most promising strategies for tissue regeneration and medical therapy (Mullenix et al., 2012).

**FIGURE 1:**
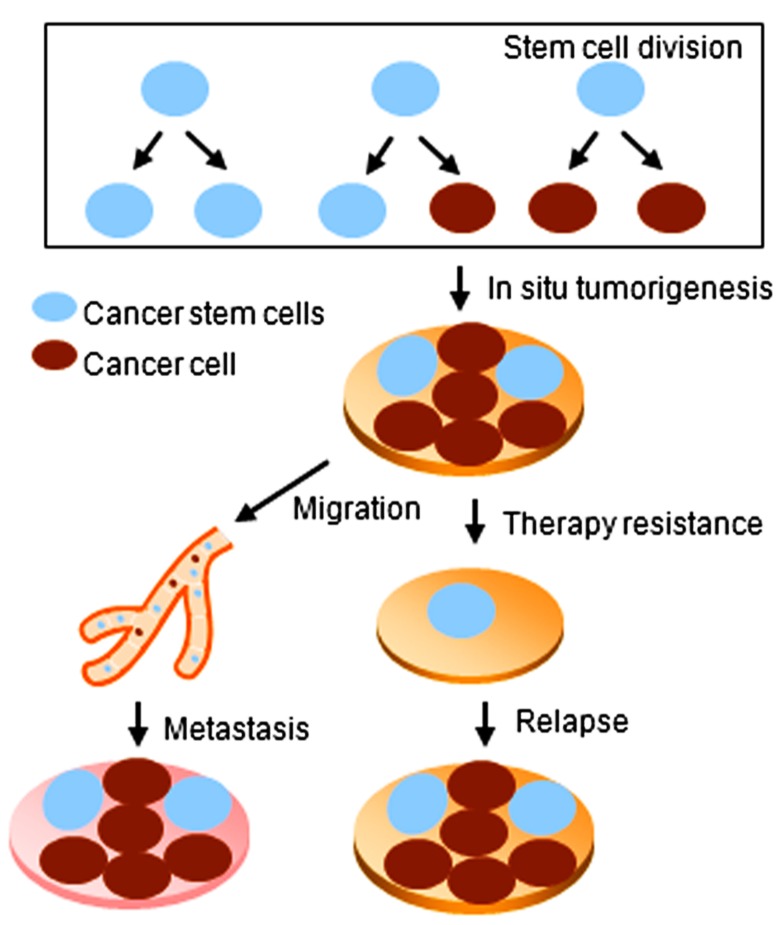
**Cancer stem cells play key roles during tumorigenesis, relapse, and metastasis of cancer**.

The concept of stem cells has been extended to the cancer field by the discovery of stem-like cells within tumors, referred to as cancer stem cells (CSCs). In contrast with ES and adult stem cells, CSCs are characterized by not only self-renewal and differentiation capacities, but also the ability to form tumors when transplanted into an animal host (Rosen and Jordan, 2009). CSCs have been isolated from different solid tumors including the breast, lung, brain, and colon (Al-Hajj et al., 2003; Singh et al., 2003; Rivera et al., 2011). CSCs are believed to be responsible for chemo- and/or radiation-therapy resistance of cancer. Moreover, CSCs may contribute to therapy relapse and tumor cell metastasis (Velasco-Velazquez et al., 2012; **Figure 1**).

Multiple signaling pathways are involved in the early cell fate decisions of ES cells, adult stem cells, and CSCs. Hormone signal transduction pathways, transcription factors, epigenetic modifications, and microRNAs (miRNAs) play important roles in regulating self-renewal and differentiation of stem cells. The epigenetic modifications include histone methylation, acetylation, phosphorylation, and DNA methylation.

miRNAs are a class of multi-functional small RNA that are single-stranded, ~22 nt in length and do not encode proteins. miRNAs regulate the stability or translational efficiency of targeted messenger RNAs through complementary interaction with the target genes. Each miRNA is predicted to target hundreds of genes. At least one-third of human mRNAs could potentially be regulated by miRNAs. miRNAs are involved in a broad range of biological processes including embryonic development, self-renewal and differentiation of stem cells, cell division and proliferation, initiation and progression of cancer, and other diseases (Sotiropoulou et al., 2009; Ye et al., 2009; Liu and Tang, 2011).

miRNAs are involved in stem cell self-renewal and differentiation by targeting components that decide stem cell fate. Following transport to the cytoplasm by Exportin-5, pre-miRNAs are processed to mature miRNAs by Dicer and its partner TBRP. Dicer-deficient mouse ES cells are defective in the capacity to self-renew or differentiate indicating the importance of mature miRNAs in ES cell self-renewal and differentiation (Kanellopoulou et al., 2005). The transcription factors OCT4, SOX2, NANOG, and KLF4 are important for the maintenance of “stemness.” These “stemness” factors are able to reprogram somatic cells to a pluripotent embryonic state (Takahashi and Yamanaka, 2006; Jaenisch and Young, 2008). miRNAs regulate the expression of these “stemness” factors. miR-145 directly represses OCT4, SOX2, and KLF4, inhibiting stem cell self-renewal and inducing differentiation (Xu et al., 2009). In a regulatory feedback loop, OCT4 in turn represses miR-145 expression (Xu et al., 2009). miR-134, miR-296, and miR-470 target the coding region sequences of Nanog, Oct4, and Sox2 genes in mouse ES cells (Tay et al., 2008). Bmi-1, a member of the Polycomb family, regulates self-renewal of stem cells by an epigenetic mechanism. Bmi-1 is activated in breast cancer stem cell (BCSC) and neural stem cell populations (Liu et al., 2006; Godlewski et al., 2008). miR-128 targets Bmi-1, suppressing CSC self-renewal and inhibiting tumorigenesis. In glioblastoma cells, Bmi-1 upregulation is associated with the downregulation of miR-128 (Godlewski et al., 2008). In addition to miR-128, miR-200, miR-203, and miR-183 target Bmi-1 thereby regulating stem cells (Shimono et al., 2009).

## STEMNESS miRNAs

Several miRNAs promote stem cell self-renewal. Expression of the miR-302-367 cluster is increased in stem cells, decreases after cell differentiation, and is undetectable in somatic cells. The miR-302-367 cluster, which includes miR-302a/b/c/d and miR-367, was originally cloned from human and mouse ES cells. The miR-302-367 cluster re-expression reprogrammed mouse and human somatic cells to a pluripotent stem cell state (Anokye-Danso et al., 2011), and reprogrammed cancer cells into an ES-like pluripotent stem cell with high expression of Oct3/4, SSEA-3, SSEA-4, Sox2, and Nanog (Lin et al., 2008). These findings suggest the miR-302-367 cluster plays a role in a cellular plasticity event by promoting “stemness” of both somatic and CSCs.

The promoter of the miR-302 cluster is regulated by Oct4 and Sox2 – two transcription factors required for stem cell maintenance (Card et al., 2008). The miR-302-362 cluster, in turn, regulates the expression of *cyclin D1* and *CDK4* – two fine-tuning regulators of G_1_/S cell cycle transition and progenitor cell function (Card et al., 2008). As such, the Oct4/Sox2-miR-302-cyclin D1 network may play an important role in maintaining the pluripotency and self-renewal properties of stem cells.

## DIFFERENTIATION miRNAs

miRNAs can induce cellular differentiation by inhibiting cell cycle transition or epithelial to mesenchymal transition (EMT), inhibiting “stemness” factors either genetic (Sox2, Oct, and Nanog) or epigenetic (Bmi-1). Several miRNAs have very low level expression in stem cells which increases upon differentiation. Expression of the let-7 miRNA family is reduced in cancer (Takamizawa et al., 2004; Yu et al., 2007). let-7 expression is very low or undetectable in BCSCs, and reduced let-7 expression is required for the maintenance of “stemness.” let-7 has high expression in differentiated cells. let-7 overexpression suppressed BCSC mammosphere formation and tumor formation in mice (Yu et al., 2007). After precession by Dicer and TBRP, mature miRNAs associate with Argonaute (Ago) to regulate mRNA expression through the RNA-induced silencing complex (RISC). Trim71, a target gene of let-7, associates with Argonaute 2 and miRNAs to repress Cdkn1a (p21^CIP1^) expression, thereby inducing the G_1_/S cell cycle phase transition to promote self-renewal of ES cells (Chang et al., 2012). let-7 delivery has been used in cancer treatment in an animal model (Esquela-Kerscher et al., 2008). miR-200c also targets the Polycomb family member Bmi-1. miR-200c inhibited mammary stem cell differentiation into mammary ducts and inhibited human BCSC tumor formation *in vivo* (Shimono et al., 2009). In breast cancer cells, miR-200 expression initiated mesenchymal to epithelial transition (MET) by targeting *ZEB1* and *ZEB2* (Eger et al., 2005). In glioblastoma, the aberrant cellular proliferation and self-renewal was associated with decreased expression of miR-128, which targets *Bmi-1* (Godlewski et al., 2008). miR-34 inhibited tumor sphere growth and tumor formation through targeting the notch signaling pathway, which is one of the most important regulators of stem cells (Ji et al., 2009).

miRNAs involved in stem cell differentiation often show reduced abundance in cancer tissues, consistent with their role as tumor suppressors. Overexpression of these miRNAs inhibits CSC self-renewal, suggesting miRNA overexpression may be useful as a therapeutic for cancer.

## EPIGENETIC MODIFICATION AND miRNA EXPRESSION

**FIGURE 2:**
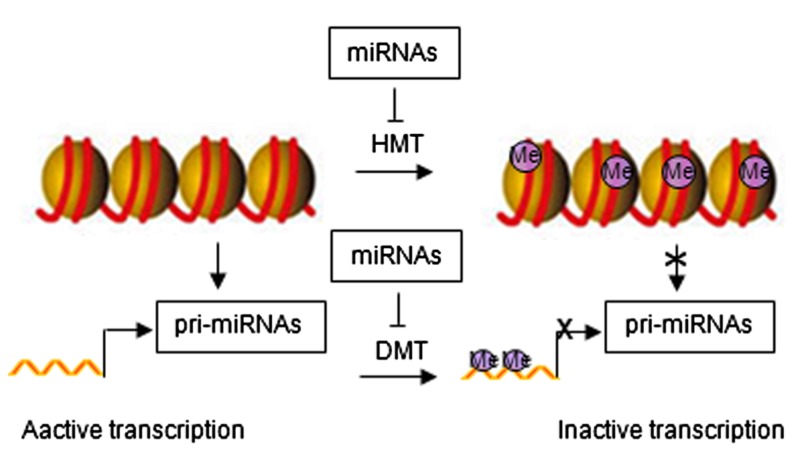
**Regulatory networks between epigenetic modification and miRNAs**. HMT, histone methyltransferase; DMT, DNA methyltransferase; Me, methylation.

Emerging evidence has identified feedback loops governing the epigenetic regulation of stem cells through miRNAs (Krutovskikh and Partensky, 2011; Liep et al., 2012). Histone modification and DNA methylation regulate expression of miRNAs. Similar to protein-coding genes, miRNAs are originally transcribed from genomic DNA, which may be affected by chromatin structure. Epigenetic modifications can alter chromatin folding resulting in the change of chromatin structure and thereby repressing or stimulating miRNA expression (**Figure 2**). Epigenetic regulation of miRNA expression has been reported in various diseases, including cancer (Saito and Jones, 2006; Lujambio et al., 2007; Liep et al., 2012). Epigenetic silencing of tumor suppressor miRNAs through CpG island promoter hypermethylation is emerging as a common hallmark of human tumors (Saito and Jones, 2006; Lujambio et al., 2007, 2008). For example, hypermethylation-related silencing of miR-148a, miR-34b/c, and miR-9 in human cancer cells is associated with lymph node metastasis (Lujambio et al., 2008). miRNA-124a undergoes transcriptional inactivation by CpG island hypermethylation in human tumors (Lujambio et al., 2007).

miRNA in turn regulate the expression of epigenetic modifiers and transcription factors through direct target interaction and/or indirect regulation of upstream pathways (Wiklund et al., 2010, 2011). For example, miR-148 can negatively regulate DNA methyltransferase expression, resulting in hypomethylation of DNA (Braconi et al., 2010). miR-449a targets histone deacetylase-1, inhibiting the expression of histone deacetylase-1, thereby inducing growth arrest of prostate cancer cells (Noonan et al., 2009).

Through these regulatory interactions, miRNAs and epigenetic modification create a feedback and/or feedforward mechanism through which gene expression is tightly controlled (Liep et al., 2012). Aberrations in these epigenetic control systems governing miRNA expression may contribute to the development of human disease.

## miRNA REGULATION OF CANCER STEM CELLS

Evidence for the function of CSCs began with human acute myeloid leukemia (AML; Lapidot et al., 1994), in which AML-initiating cells were identified from patients on the basis of the cell surface marker CD34^+^^+^CD38^-^ expression. Upon transplantation into severe combined immune-deficient (SCID) mice, this cell population homed to the bone marrow and showed a pattern of dissemination and leukemic cell morphology similar to the original patients. Subsequently, CSCs have been identified in a variety of solid tumors, including breast, brain, colon, pancreas, lung, prostate, liver, melanoma, glioblastoma, and head and neck cancer. The CSCs have been defined by expression of distinct tissue type-specific cell surface marker including CD44, CD24, and CD133 (Al-Hajj et al., 2003; Singh et al., 2003).

Cancer stem cells undergo asymmetric cell division to maintain both a stem cell and a differentiated cancer cell population. As CSCs are often resistant to chemotherapy and/or radiation-therapy, CSCs are thought likely to contribute to tumor recurrence. miRNAs are involved in the regulation of CSC self-renewal and differentiation (Bartel, 2004; Shimono et al., 2009). A CSC-specific miRNA expression profile has been reported (Shimono et al., 2009) and altered expression of miRNAs has been identified in cancer stem/ progenitor cells which vary by tumor type (Qian et al., 2008; Shimono et al., 2009). A recent report defined distinct miRNA expression patterns in various stem/progenitor cell populations in prostate cancer, demonstrating the downregulation of tumor suppressor miRNAs including miR-34 and let-7 in prostate CSCs (Liu et al., 2012), which revealed a coordination of miRNAs in regulating CSC self-renewal and cancer cell proliferation.

## CONCLUDING REMARKS

In view of the changes in miRNA abundance in a range of human diseases including cancer, miRNAs are expected to be targeted for therapy. Synthetic miRNA mimics and DNA expression vectors containing miRNA precursor sequences have strong potential as therapeutic tools. Although the therapeutic trial of miRNA mimics or expression vectors in animal models has been reported to suppress tumor growth (Esquela-Kerscher et al., 2008; Kota et al., 2009), the ideal delivery system with the characteristics of high transfection efficiency, low cellular toxicity, protection of miRNAs from degradation, and tissue-specific delivery remains a challenge. The side effects of miRNA therapy to date result from either off-target tissue effects in other organs or altered gene expression from off-target gene effects as miRNA targets multiple genes. In addition, the onco-miRNAs make them excellent targets for cancer drug treatment. Chemically modified miRNA antisense oligonucleotides have successfully inhibited target miRNA *in vitro* (Zhu et al., 2007) and *in vivo* (Garchow et al., 2011). Using this technique, a new anti-cancer approach targeting the oncogenic miRNAs is being adapted for clinical application. Interestingly, the discovery of circulating miRNA in body fluids including serum suggests miRNAs may have the potential to serve as diagnostic or prognostic biomarkers for human disease (Gilad et al., 2008; Kosaka et al., 2010).

**FIGURE 3:**
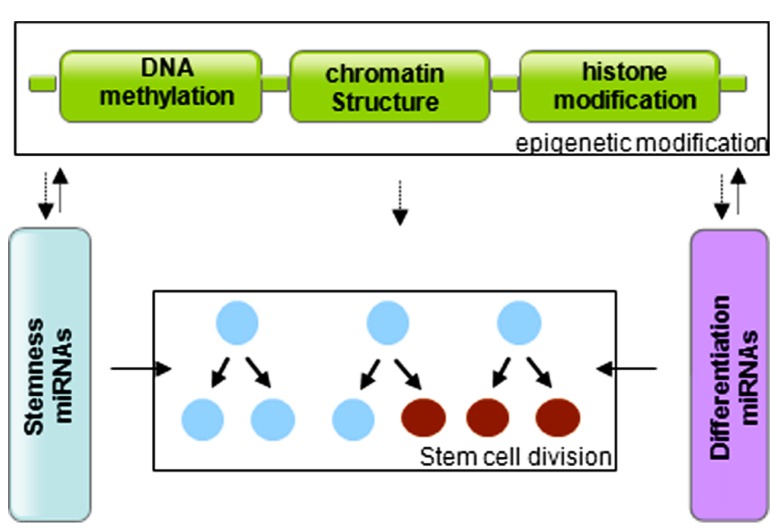
**Graphic of the reciprocal association between microRNAs and epigenetic modifications in control of stem cell division**.

In summary, stem cells are controlled by genetic and epigenetic regulatory networks that maintain the balance between self-renewal and differentiation. The epigenetic regulation includes a variety of modifications that affect DNA methylation and histone modification. miRNAs regulate the stem cell characteristics through regulating the expression of transcription factors. miRNA genes are in turn subjected to epigenetic modification, and miRNAs themselves can modify chromatin structures (**Figure 3**). The cooperative association and reciprocal interactions between genetic and epigenetic regulatory factors and miRNAs regulate the self-renewal and differentiation of stem cells, and/or reprogramming of differentiated cells to induced pluripotent stem cells.

## Conflict of Interest Statement

The authors declare that the research was conducted in the absence of any commercial or financial relationships that could be construed as a potential conflict of interest.
